# Social structure contains epidemics and regulates individual roles in disease transmission in a group‐living mammal

**DOI:** 10.1002/ece3.4664

**Published:** 2018-11-11

**Authors:** Carly Rozins, Matthew J. Silk, Darren P. Croft, Richard J. Delahay, Dave J. Hodgson, Robbie A. McDonald, Nicola Weber, Mike Boots

**Affiliations:** ^1^ Department of Integrative Biology University of California, Berkeley Berkeley California; ^2^ Centre for Ecology and Conservation University of Exeter Penryn, Cornwall UK; ^3^ Environment and Sustainability Institute University of Exeter Penryn, Cornwall UK; ^4^ Centre for Research in Animal Behaviour, College of Life and Environmental Sciences University of Exeter Exeter UK; ^5^ National Wildlife Management Centre Animal and Plant Health Agency Gloucestershire UK

**Keywords:** contact network, European badger, infectious disease, initially infected, modularity, network weighting

## Abstract

Population structure is critical to infectious disease transmission. As a result, theoretical and empirical contact network models of infectious disease spread are increasingly providing valuable insights into wildlife epidemiology. Analyzing an exceptionally detailed dataset on contact structure within a high‐density population of European badgers *Meles meles,* we show that a modular contact network produced by spatially structured stable social groups, lead to smaller epidemics, particularly for infections with intermediate transmissibility. The key advance is that we identify considerable variation among individuals in their role in disease spread, with these new insights made possible by the detail in the badger dataset. Furthermore, the important impacts on epidemiology are found even though the modularity of the Badger network is much lower than the threshold that previous work suggested was necessary. These findings reveal the importance of stable social group structure for disease dynamics with important management implications for socially structured populations.

## 1 INTRODUCTION

Sociality is widespread in animal populations (Couzin & Laidre, 2009; Hirth, 1977; Krause & Ruxton, 2002; Macdonald, 1983) and has important implications for the epidemiological dynamics of host–pathogen relationships because it prevents random‐mixing among individuals. In many populations, there is considerable heterogeneity in social contacts capable of transmitting infections among members (Craft, 2015; Lloyd‐Smith, Schreiber, Kopp, & Getz, 2005; VanderWaal & Ezenwa, 2016; White, Forester, & Craft, 2017). Highly connected individuals have received particular attention as they play a pivotal role in a number of important human diseases (Lloyd‐Smith et al., 2005). However, the role of these highly connected individuals in wildlife populations remains uncertain, especially for species that tend to live in social groups. The occurrence of spatial or social community structures within a population result in a social network with high modularity (whereby intra‐group interactions predominate over inter‐group interactions; Newman, 2002; Sah, Méndez, & Bansal, 2018). Populations with more modular social networks typically experience smaller and slower‐spreading epidemics (Griffin & Nunn, 2012; Miller, 2009; Newman, 2003; Salathé & Jones, 2010; Shang, Liu, Li, Xie, & Wu, 2015). This reduction in disease spread occurs because high fragmentation and close‐knit subgroupings delay the spread of disease and serve to “trap” infections within networks (Sah, Leu, Cross, Hudson, & Bansal, 2017). It is also likely that the role of individuals in the spread of disease may change in more modular networks, whereby individuals that act as “bridges” between different regions of network will be integral to regulating disease transmission (Salathé & Jones, 2010). However, the importance of these bridging individuals in wildlife populations made up of multiple stable social groups has received little attention.

Previously networks derived from empirical observations (Rushmore et al., 2014; VanderWaal, Atwill, Isbell, & McCowan, 2014) have been used to provide insights into the role of network structure in disease transmission in nonhuman animals. However, recent advances in bio‐logging technology to collect high‐resolution social contact data, and methods of network analysis, have enabled the quantification of social interactions among wild animals (Blyton, Banks, Peakall, Lindenmayer, & Gordon, 2014; Hamede, Bashford, McCallum, & Jones, 2009; Hirsch, Reynolds, Gehrt, & Craft, 2016; Pinter‐Wollman et al., 2013; Weber, Carter, et al., 2013; White et al., 2017). This has facilitated modeling work that has provided many important insights into how social systems and network structure influences the transmission of directly transmitted infections in nonhuman animals (e.g., Sah et al., 2018). However, an important gap remains in understanding the role of stable social group structure at a population level (cf. work within groups; Nunn, Craft, illespie, Schaller, & Kappeler, 2015; Sah et al., 2018; VanderWaal et al., 2014; Weber, Carter, et al., 2013; White et al., 2017), and in particular how it interacts with the role of individuals in the spread of infection.

**Figure 1:**
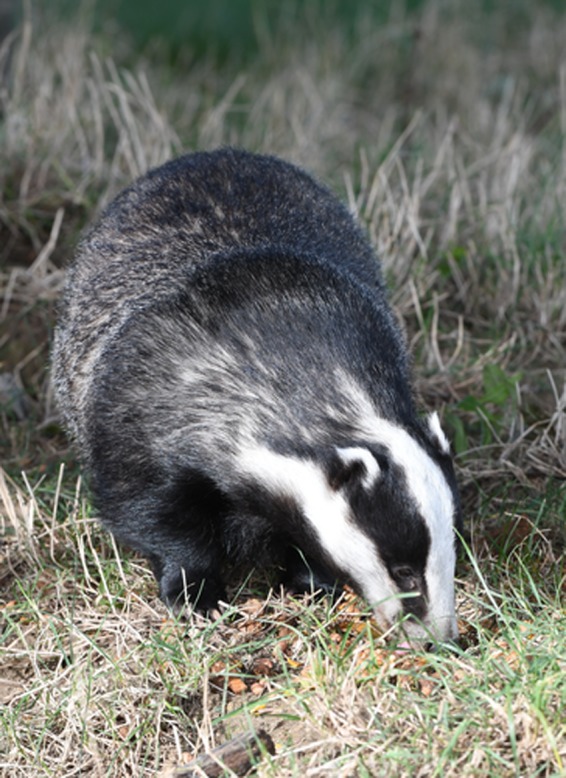
European badger, *Meles meles*

We exploit a contact network dataset collected in a high‐density population of European badgers *Meles meles* (Figure 1) in Gloucestershire (UK) using UHF proximity loggers (Sirtrack Ltd, Havelock, New Zealand). In much of the United Kingdom and Ireland, badgers live at higher densities than in the rest of the species’ range (McDonald, Robertson, & Silk, 2018; Roper, 2010); they live in territorial social groups that share communal dens known as setts (Roper, 2010). Individuals in these populations interact very frequently with others from the same group but much more sporadically with those from neighboring groups (Roper, 2010; Weber, Carter, et al., 2013). This results in modular contact networks, in which individuals from the same social group are much more closely connected than individuals from different social groups. While there are some differences in social contacts over the course of a year (Silk et al., 2017), the overall structure of the networks persists.

We used disease simulations to examine the implications of social group structures for both the risk and size of epidemics in networks generated directly from the empirically derived European badger contact network (Weber, Carter, et al., 2013). We also investigate the impact, on spread of infection and epidemic size, of (a) the duration of contact, (b) identity, and (c) social position of the first‐infected individual. We simulated the spread of a generic infection with SIR (susceptible‐infected‐removed) type dynamics for a range of transmission probabilities (and subsequently a range of basic reproductive ratios—*R*_0_).

## 2 METHODS

### 2.1 Empirical data collection

Data were collected from a high‐density population of badgers in Woodchester Park, Gloucestershire, UK. This population has been the subject of a long‐term mark–recapture study since the 1970s (Delahay et al., 2013; McDonald et al., 2018). A detailed capture history is available for all individuals in the population. Data for the social networks used in this study were collected by using proximity‐logging radio tags (Sirtrack) to capture the interactions between 51 individuals living in eight communal setts located at the core of this long‐term study population. Data were collected over a 1‐year period from June 2009 to May 2010 (Weber, Carter, et al., 2013). Individuals used to construct the networks included subadults and adults, consisted of 24 males and 27 females, and represents 80% of the total population.

### 2.2 Generating simulated networks

Simulation of networks based on the observed dataset allowed us to incorporate uncertainty in epidemiological estimates. We simulated single (static) annual networks as the network structure is qualitatively similar throughout the year, and this approach enabled us to incorporate all of the information we had on the contacts of individuals. Networks were simulated that (a) matched the spatial structure of the observed network data (referred to as spatially structured networks SSN), (b) matched the degree distribution (individual variation in number of connections in the network) but not spatial structure of the observed network (referred to as spatially unstructured networks SUN), and (c) were random networks with identical density (number of edges) to the empirically derived network (referred to as random unstructured networks RUN). All simulated networks comprised 51 nodes, the same as the number of badgers in the observed network.

**Figure 2:**
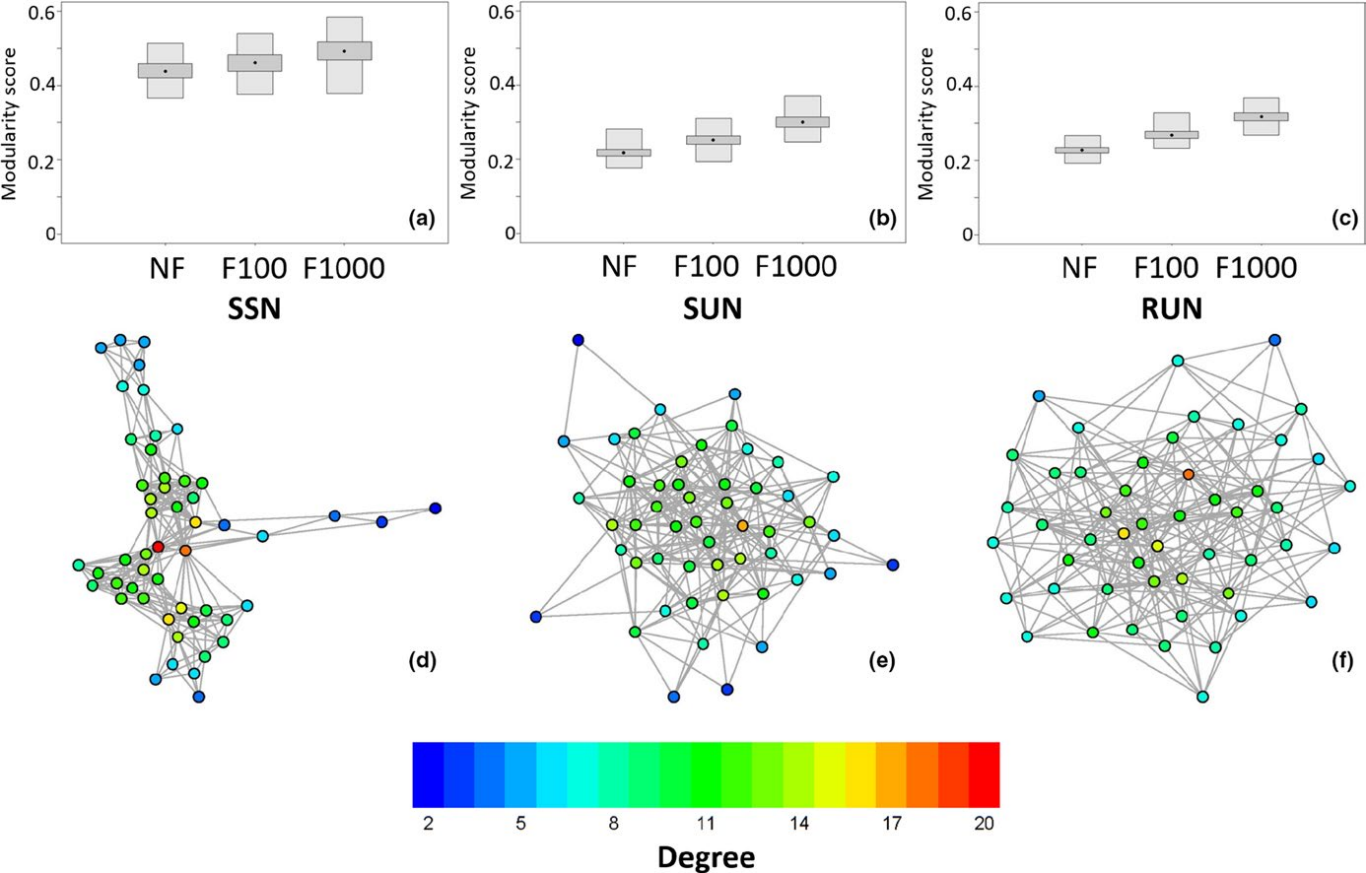
Network structure of the observed and simulated networks used in this study: (a) the distribution of modularity scores for networks simulated with equivalent social group and spatial structure (SSN), (b) the distribution of modularity scores in spatially unstructured networks SUN, (c) the distribution of modularity scores in random networks (RUN), (d) the structure of the empirical badger contact network, (e) an example of a simulated unstructured network (SUN), and (f) an example of a simulated random network (RUN). Nodes in each network are colored according to their degree and each boxplot is based on 1,000 generated networks, all modularity scores were calculated using the fast greedy algorithm in R package igraph and weighted edges have been transformed to binary according to set temporal cut‐offs (NF: all edges are included, F100: only edges with contact durations >100 s are included and F1000: only edges with contact durations >1,000 s are included)

Spatially structured networks were simulated from the observed network data collected by Weber, Bearhop, et al. (2013 and Weber, Carter, et al., 2013). The observed association data were fitted with a zero‐inflated negative binomial generalized linear model in the R package pscl (Zeileis, Kleiber, & Jackman, 2008). The duration (in seconds) of interactions between every dyad in the population was the response variable in the model. The explanatory variables were the distance in meters between the main setts in which two individuals were caught, the distance in terms of social group territories between two individuals and whether or not two individuals were the members of the same territorial group (as a binary indicator variable) according to bait marking studies (Delahay et al., 2000) completed in 2009. Using this method, networks were simulated that closely matched the observed network in mean degree (Supporting Information Table S1), degree distributions and “spatial” structure/modularity (Figure 2).

We also generated spatially unstructured networks (SUN) with the use of the rewire() function in the R package igraph (Csardi & Nepusz, 2006) which generated equivalent networks with equal degree distribution but without “spatial” structure for each simulated. Finally the function random.graph.game() in igraph was used to generate random networks (RUN) with the equivalent number of edges to the binary full networks.

Following Sah et al. (2017), we analyzed unweighted (binary) versions of these networks. This is a conservative approach as by ignoring the weights of edges the modularity of the observed social structure is reduced (i.e., it assigns all connections as being equal; therefore, weak between group connections are treated as equally important as strong within group connections). An additional advantage of this approach, rather than including the contact duration as a parameter in disease transmission (see Rushmore et al., 2014), is that it allows for stochastic variability in individual susceptibility to disease transmission. In order to confirm that our results would be similar for infections that required longer contact durations, we repeated our main analyses in (a) unfiltered networks including all edges (NF), (b) networks filtered to include only edges with contact durations >100 s (F100), and (c) networks filtered to include only edges with contact durations >1,000 s (F1000). Finally, using the R package igraph (Csardi & Nepusz, 2006), network community structure and modularity scores were determined. We used the fast greedy algorithm to define social communities, and report information for the number of communities with the highest modularity score.

### 2.3 Network measures

Three measures of individual connectedness (centrality) were calculated for all of the nodes on each of the unfiltered simulated networks: degree (number of edges), eigenvector centrality (rewards edges/connections to important nodes), and betweenness centrality (rewards nodes that act as bridges between other nodes). Betweenness centrality and eigenvector centrality were calculated in R using the packages igraph (Csardi & Nepusz, 2006) and sna (Butts, 2007) and the functions betweenness() and eigen_centrality(), respectively.

### 2.4 Epidemic simulation

All simulations started with a single infected individual in a completely susceptible population. Following (Sah et al., 2017), all simulations follow an SIR‐type (Susceptible‐Infected‐Removed) model where individuals fall within one compartment, they are either susceptible to infection, infectious, or removed. All 51 individuals were chosen to be patient zero 100 times. Therefore, for a network of a particular class (SSN, SUN or RUN), we ran 5,100 simulations for nine unique transmission probabilities *T*, on 1,000 unique graphs. Transmission probabilities were chosen to be between 0.075 and 0.275.

Simulations proceeded as follows:

1. A single individual is infected (patient zero).
2. Within the first iteration, patient zero's neighbors (those connected by an edge) become infected (and infectious) with probability *T* and patient zero is removed from the network.
3. In all subsequent iterations, the neighbors of any infectious individual become infected (and infectious) with probability *T*, and all previously infectious individuals are removed from the network.

Note that the iterative steps do not explicitly represent uniform time units, but follow a bond percolation‐like approach. This approach, one that ignores temporal dynamics, has been shown to produce similar results to time‐sensitive simulations (Rushmore et al., 2014), but is less computationally expensive. The disease will work its way through the network as described above, until no new individuals can become infected. The termination of an outbreak can be the result of it burning out due to a lack of successful transmission events, or because there are no more susceptible individuals to infect.

### 2.5 Basic reproduction number and the epidemic threshold

For each network, we determine the basic reproduction number: *R*_0_ = T(<k^2^>/<k>−1), where, <*k*>, is the mean degree, <*k* ^2^>, is the mean squared degree and, *T,* the transmission probability (Newman, 2002). This calculation of *R*_0_ will produce identical *R*_0_ values for networks of identical degree (i.e., SSN and SUN networks). The epidemic threshold refers to the transmission probability for which *R*_0_ = 1. Therefore, for each transmission parameter and network, we compute the corresponding value of *R*_0_, which is more easily interpreted epidemiologically than simply a transmission probability (Supporting Information Table S2).

### 2.6 Statistical analysis

#### 2.6.1 Variation in epidemic size given the identity of the initially infected individual

We used data from disease simulations to quantify the variation in (a) the number of secondary infections and (b) the epidemic size, given the identity of the initially infected individual, for the three different network types (SSN, SUN, RUN). This involved calculating the proportion of variation explained by the initially infected individual (c.f. stochastic variation in the number of secondary infections and outbreak size) using the R package rptR (Schielzeth & Nakagawa, 2011). To investigate variation in epidemic size a proportional, binomial generalized mixed effects model (GLMM) was used with an intercept and individual as the only random effect. A similar Poisson GLMM was used to calculate repeatability for the number of secondary infections. Repeatabilities were calculated separately for each transmission probability in each of the 1,000 networks generated. This made it possible to determine whether transmission probabilities affected the proportion of variation explained by the choice of the initially infected individual, and provided a distribution of 1,000 repeatability scores for each combination of network type and transmission probability. Additionally, in order to demonstrate the importance of the interaction between transmission probability and the type of network in the repeatability of outbreak size and the number of secondary infections we fitted seven competing Bayesian linear models in Stan using the R package brms (Burkner, 2017) in R 3.3.2 and compared the strength of support for each model with Watanabe–Akaike information criterion (WAIC) scores of the models.

#### 2.6.2 Epidemic size as an outcome of the network position of the initially infected individual

We used data on the mean epidemic size for each individual in the unfiltered networks to quantify the effect of degree, eigenvector centrality, and betweenness centrality on the size of the epidemic separately for each transmission probability in each type of network. Mean epidemic size was divided by the total size of the network (to produce a mean proportion of the population infected), and then, the logit function was used to transform this variable. First we examined each centrality measure in separate linear mixed effects model to assess differences in their power in predicting epidemic size. We included a network measure as a fixed effect variable alongside a random effect to control for network identity (including both a random intercept and random slope). Due to the expectation that the effect of centrality on epidemic size might decline for larger centrality values (especially for infections with higher transmission probabilities), we fitted two models for each centrality measure, one in which the raw values of the measure were used, and one which had been log(Measure+1) transformed. Second we constructed a combined linear mixed effects model which included fixed effect variables of all three centrality measures after they had been log(Measure+1) transformed. This model included a random intercept and uncorrelated random slopes for each network measure related to the identity of the network. This second model enabled us to identify the importance of indirect connections (measured using eigenvector centrality and betweenness centrality) while controlling for the effect of direct contacts (degree).

## 3 RESULTS

The observed badger social network displayed clear community structure (Figure 2d). The weighted contact network was formed of six communities with a modularity score of 0.462 for this division. The binary contact network was split into three communities with a modularity score of 0.484 for this division. Networks simulated using a negative binomial function fitted to the observed dataset (SSN; see methods) retained this modular structure (Figure 2a), as well as having a similar mean (unweighted) degree (Supporting Information Table S1) and (unweighted) degree distribution (Supporting Information Figure S1). Networks rewired to maintain the degree distribution without retaining the spatial and social group structure of the original network (SUN; Figure 2b) and random networks (RUN; Figure 2c) had considerably reduced modularity (Figure 2). Networks filtered to only contain edges of longer durations had higher modularity scores than unfiltered networks (Figure 2). The epidemic threshold, calculated by setting *R*_0_ = 1, was greater in the random network then the structured networks (SSN, SUN), regardless of filtering level (NF, F100, F1000; Supporting Information Table S3). Additionally, as filtering increased, so too did the epidemic threshold. Thus, highly filtered networks (F1000) require a more intense infection (higher transmission probability, *T*) for an epidemic to occur.

**Figure 3:**
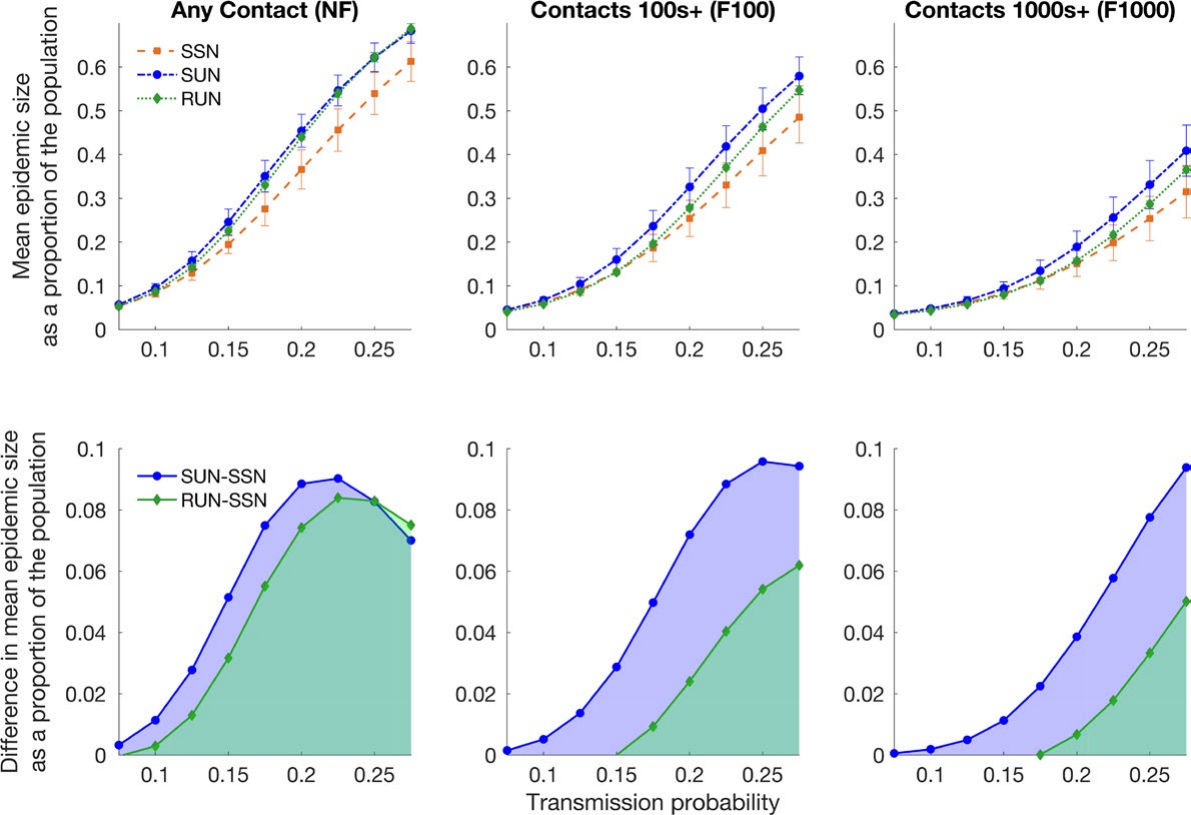
Mean epidemic size for the spatially structured network (SSN), spatially unstructured network (SUN) and random network (RUN). Interquartile error bars are calculated from the mean epidemic size per individual after 5,100 simulations (100 per individual) for each of the 1,000 unique networks, for each of the edge‐weight filtering levels (NF, F100, F1000). Transmission probabilities are between 0.075–0.275 and 5,100,000 simulations were run for each transmission probability, for each network type

**Figure 4:**
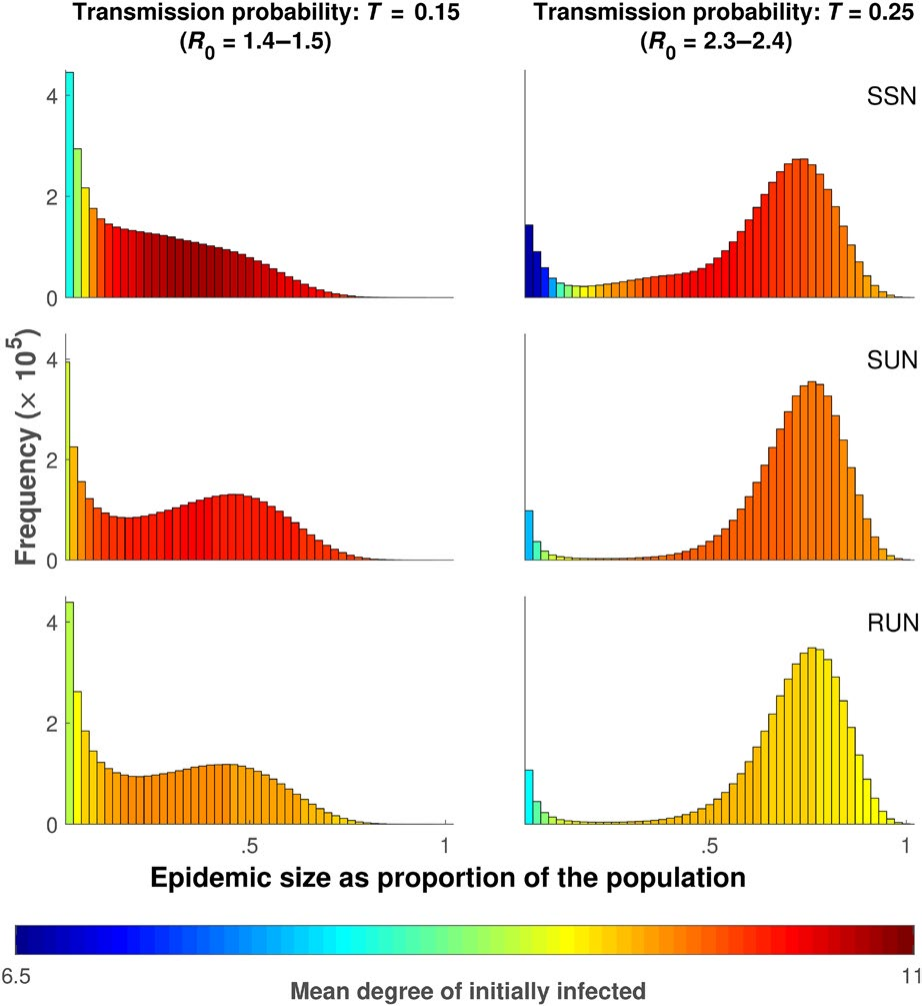
Distribution of epidemic sizes for simulations in unfiltered networks (NF) where the transmission probability is held at *T* = 0.15 (left) and for simulations in which the transmission probability is held at *T* = 0.25 (right). The epidemic size is on the *x‐*axis (as a proportion of the total population) and the frequency (the number of times an epidemic of that size occurred) is on the *y‐*axis. Each individual plot represents results from 5,100,000 simulations. The 100 simulations for each initially infected individual were not averaged, but kept as a single simulation result. The color of the individual bar represents the mean degree of the initially infected individual that resulted in an epidemic of that size. The larger *R*_0_ values (1.5 and 2.4) are the values for the networks that share the same degree distribution (SUN and SSN) while the smaller value (1.4 and 2.3) are for the RUN

Simulated epidemic outbreaks in realistically spatially and socially structured networks (SSN) were smaller on average (Figures 3 and 4; Supporting Information Figure S2) and considerably less likely to reach the average epidemic size found in spatially unstructured (SUN) or random networks (RUN; Figures 3 and 4, Supporting Information Figure S2). The difference in mean epidemic size, between the SSN and the SUN or RUN, peaked for a transmission probability of *T* = 0.225 (*R*_0_ = 2.21), for the unfiltered networks, (NF). For filtered networks (F100 and F1000), a similar pattern was evident but the peak difference in mean epidemic size occurred at higher transmission probabilities as would be expected for a less well‐connected network. The peak difference occurred at *T* = 0.25 (*R*_0_ = 2.45) for the F100 networks and had not occurred by *T* = 0.275 (*R*_0_ = 2.7) for the F1000 networks. The effect of transmission probability on outbreak size was strongest for unfiltered networks (NF; Supporting Information Table S6 and S8).

**Figure 5:**
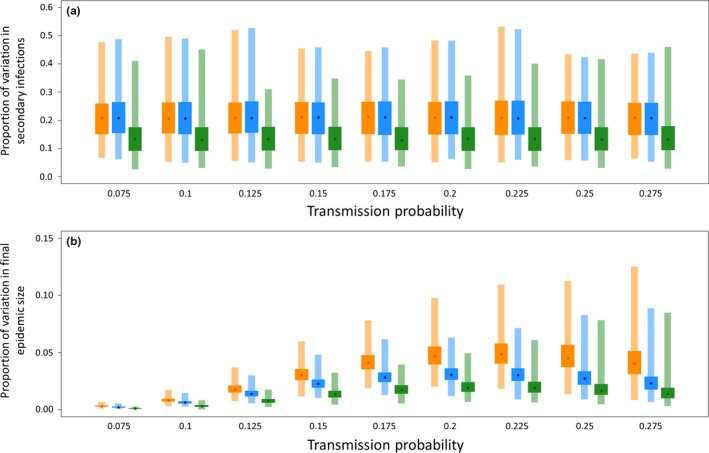
(a) The proportion of variation in (a) the number of secondary infections and (b) epidemic size explained by the choice of the initially infected individual in the three network types for each transmission probability used in the study. Orange is the structured network (SSN), blue is the unstructured network (SUN), and green is the random network (RUN). Points represent the median, wide boxes the interquartile range and narrow boxes the range of values calculated from each of the 1,000 simulated networks of each type

There were also important differences between the three network types in the variation in the number of secondary infections (i.e., the number of individuals infected directly by the initially infected individual) and epidemic size (Figure 5, Supporting Information Table S6 and S7) explained by the selection of the initially infected individual. More variation in the number of secondary infections was explained by the social position of the initially infected individual in spatially structured (SSN) and spatially unstructured networks (SUN) than in random networks (RUN), and this was independent of transmission probability (Figure 5a, Supporting Information Table S7). This similarity between SSN and SUN is expected because they have identical degree distributions. However, in spatially structured networks (SSN), more variation in epidemic size was explained by the choice of the initially infected individual than either spatially unstructured (SUN) or random networks (RUN; Figure 5b, Supporting Information Figure S3 and S4). For networks that retained all edges (NF), we found that the importance of the identity of the initially infected individual was greatest for intermediate transmission probabilities (Figure 5b). However, for filtered networks (F100, F1000), the transmissibility (and hence *R*_0_) of the pathogen had no impact on the degree of variation (Supporting Information Figure S3 and S4). Therefore, in networks with realistic social and special structuring, there is greater individual heterogeneity in spreader status compared with networks that lack this structuring. This reveals that heterogeneity in importance for disease transmission can be greatest in modular networks without “superspreader” dynamics, and that the role of heterogeneity can vary among pathogens with different levels of infectiousness.

**Figure 6:**
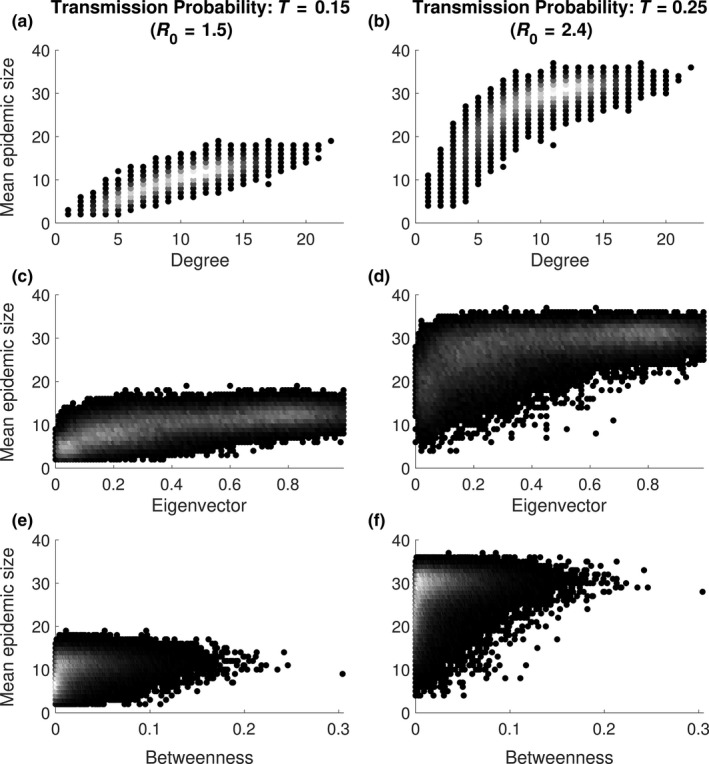
Mean epidemic size given the (a–b) degree, (c–d) eigenvector centrality and (e–f) betweenness centrality, of the initially infected individual for disease simulations on the unfiltered (NF), spatially structured network (SSN), for two transmission probabilities. The coloring reflects the frequency of observations, with lighter colors reflecting a higher frequency and darker colors lower frequency

For all types of networks the epidemic size increased with increasing degree (number of direct connections) of the initially infected individuals, with the average epidemic size increasing as the degree of the initially infected individual increased (Figure 6; Supporting Information Tables S4 and S5). This effect was somewhat linear for the lowest transmission probabilities, but reached an asymptote when transmission probabilities were high (Figure 6). Similar positive effects of other centrality measures that incorporated indirect connections were also found, but these were considerably weaker than those for degree and did not persist in random networks (Supporting Information Tables S4). For all centrality measures at all transmission probabilities, a log‐linear relationship provided a better fit to the simulation data than a linear relationship in realistically structured badger networks (Supporting Information Tables S5), indicating that even for more slowly spreading pathogens, the rate of increase in epidemic size with centrality was higher for less central individuals.

**Figure 7:**
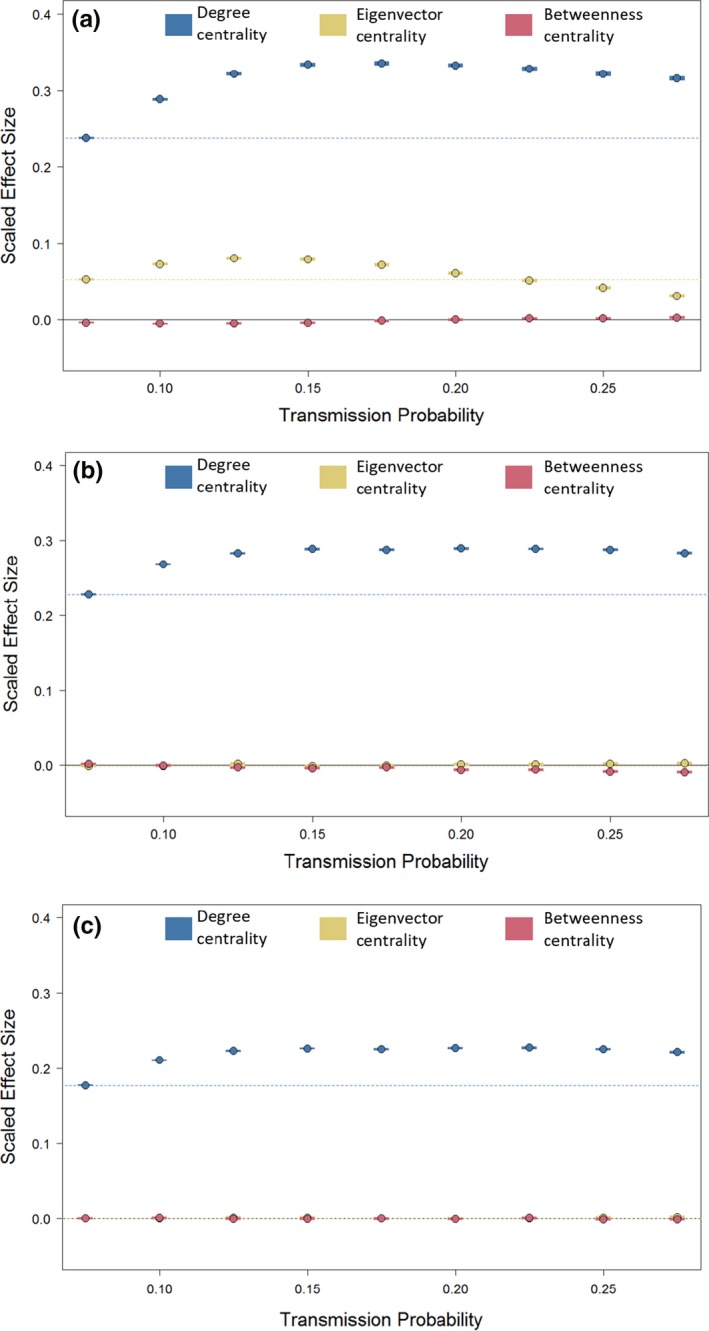
The effect of increasing different centrality measures on mean epidemic size in (a) spatially structured networks (SSN), (b) spatially unstructured networks (SUN) and (c) random networks (RUN). Points represent model estimates from model predictions and shaded areas represent the estimate ±95% confidence intervals (blue: degree, yellow: eigenvector centrality, pink: betweenness centrality). Dashed lines represent the model prediction for the effect of degree (blue) and eigenvector centrality (yellow) at the lowest transmission probability to facilitate comparisons. Effect sizes are from a model including all three measures so represent the effect of each measure while accounting for variation in the others. All centrality measures have been log(Measure+1) transformed and then scaled to be mean centered and have unit variance

When the effect of all three of the centrality measures were considered within the same model, there were important differences between the spatially structured networks and the spatially unstructured and random networks with regard to the importance of indirect connections (Figure 7). In the random networks, neither eigenvector centrality nor betweenness centrality had an additional effect to degree on epidemic size at any transmission probability (Figure 7c). For spatially unstructured networks, eigenvector centrality had no additional effect on transmission probability and the effect of betweenness was very limited, being slightly negative for infections with higher transmission probabilities (Figure 7b). However, in realistic spatially structured networks, both eigenvector centrality and betweenness centrality influenced epidemic size in addition to the effect of degree (Figure 7a). Eigenvector centrality had an intermediate positive effect that peaked for infections with intermediate transmission probabilities and was lowest for infections with high transmission probabilities. Betweenness centrality had a weak effect that was opposite to its effect in spatially unstructured networks. Higher betweenness resulted in slight reductions in mean epidemic size for slow spreading infections and slight increases in mean epidemic size for highly transmissible infections while controlling for the values of other metrics.

## 4 DISCUSSION

Our disease simulations showed that the spatial structure of empirically derived badger contact networks reduced the probability of large epidemics. This provides evidence for the importance of a “social bottleneck” (Nunn, Craft, et al., 2015; Sah et al., 2018; VanderWaal et al., 2014; Weber, Carter, et al., 2013; White et al., 2017) in disease transmission functioning at a population level. We also found considerable variation between individuals in the size of epidemics they generated in networks with realistic spatial and social group structure that varied according to both their local (direct connections) and global position (role in broader network connectivity) within the social network. Importantly, the impact of indirect connections on the role of an individual in disease spread depended upon the transmissibility of the pathogen involved.

Our results agree with evidence from other simulation studies, that networks with higher modularity will have smaller epidemics and lower peak prevalence of infection (Newman, 2003; Salathé & Jones, 2010). The most modular networks in this study were those that were both spatial and socially structured. Interestingly, networks with identical social structure (degree distribution), but lacked spatial structure, have lower modularity and higher mean outbreak size, even when compared to random networks. This suggests that social connections, in the absence of spatial structure, promote disease spread. Our results highlight that the importance of community structure extends to much lower levels of modularity (Q ~ 0.45) than the high modularity scores used by the most directly comparable previous study (Salathé & Jones, 2010). Previous simulation models have found that groups with structure (and therefore higher modularity) tend to have lower parasite prevalence due to the presence of social bottlenecks (Nunn, Jordán, McCabe, Verdolin, & Fewell, 2015; VanderWaal et al., 2014). At a population level, social bottlenecks would be expected to result in the aggregation of infection within particular social groups for pathogens with low to intermediate transmission probabilities (Manlove, Cassirer, Cross, Plowright, & Hudson, 2014). By exploring the effect of modularity for a greater range of transmission probabilities, we were able to reveal that the effect of community structure varies, depending on the transmissibility of the pathogen being investigated, as well as the duration of contacts being considered. While we found a strong relationship between modularity and epidemic size, it should be noted that other network properties might change along with modularity that we do not account for. However, it would not be possible to completely change the modularity of a network while maintaining identical values for all other higher order properties of the network (such as degree distribution). Therefore, disentangling these properties, while remaining biologically meaningful, is difficult.

The reduction in epidemic size was greatest for pathogens with intermediate rather than low or high transmission probabilities (*R*_0_ = 2.2) when all contacts, regardless of duration, were considered capable of transmitting infections. This suggests that the impact of structural delay and trapping of infection spread that is apparent in modular networks (Sah et al., 2017) peaks for infections that are able to spread effectively, but do not have sufficiently high transmission probabilities to facilitate escape from subregions of the network (Cross, Lloyd‐Smith, Johnson, & Getz, 2005). The importance of social structure in trapping infection further supports the idea that badgers that form more out‐of‐group contacts can act as “capacitors” in controlling the spread of infection under certain conditions (Weber, Carter, et al., 2013), especially for less readily transmissible pathogens. Additionally, populations with highly modular networks often consist of a large proportion of both highly central, as well as highly isolated individuals (characteristic of high eigenvector centralization; Griffin & Nunn, 2012). For highly transmittable infections, the isolated nodes can act to limit the outbreak size, whereas for lower transmissible infections, the highly central individuals may drive an outbreaks.

The contact duration that permitted transmission of infection (i.e., level of edge‐weight filtering in disease simulations) had a strong impact on the size of the epidemic for a given transmission probability. However, it did not alter the importance of modularity in limiting epidemic size compared to networks without spatial structure, tending to simply mean that the peak difference occurred for simulations with higher pathogen transmissibility. In fact, the increased modularity of networks that only included long duration contacts meant that the modularity of the socially structured contact network had a greater limiting effect on the size of an epidemic. Therefore, it is likely that understanding what types or durations of social interactions are required to transmit an infection will be integral to assessing the impact of modular network structures on disease spread in wildlife populations in addition to knowledge of the *R*_0_ of a given pathogen.

Considerable variation in the number of secondary infections and the epidemic size was explained by the choice of initially infected individual. However, this variation does not result in what might be considered conventional superspreader dynamics, in which epidemics are less likely but larger in size (Lloyd‐Smith et al., 2005). Rather, our findings suggest the opposite, with epidemics being just as likely but smaller in size in networks with realistic spatial and social structure. Further, the most severe epidemics in networks with realistic social structure did not necessarily stem from initially infected individuals with the highest degree, and depended in part on other aspects of network position that accounted for indirect connections. This emphasizes the potentially conflicting effects of high modularity and heterogeneity in network position on disease dynamics. The relationship between superspreader‐type dynamics and the limiting effects of spatial and social structure may therefore be fundamental in driving the dynamics of host–pathogen interactions in natural populations. Finally, the individual network measures, and their impact on the epidemic size, are likely to hold true in the complete badger social network (recall the 51 badgers make up 80% of the total population). While concerns have been expressed on the reliability of using social networks constructed using a subset of the population, it has been found that an individuals social and spatial importance should not change with the addition of individuals to the network (Silk, Jackson, Croft, Colhoun, & Bearhop, 2015).

Culling of high‐density badger populations has been used as an attempt to control disease, but has proved controversial (Mcdonald, 2014). It has been postulated that culling‐induced perturbation of the badger social system comprising increased ranging behavior, less clearly defined territorial boundaries and increased dispersal has reduced the effectiveness of this approach (Carter et al., 2007; McDonald, Delahay, Carter, Smith, & Cheeseman, 2008). By demonstrating the importance of host social structure in limiting epidemic size at a population level, our results provide a novel insight into how social perturbation of badger populations might be detrimental from a disease control perspective; decreasing the modularity of the social contact network may be integral to increases in disease incidence and epidemic size that can result from social perturbation. Social perturbation has been suggested to be problematic for disease control in wildlife hosts (Laddomada, 2000; McDonald et al., 2008), and the lessons learned could be applied more generally in cases where animal social networks are naturally modular (Weber, Carter, et al., 2013) to identify situations where social perturbation might have particularly important consequences for pathogen transmission. For example, social group structure seems likely to be important in limiting epidemic size in many species, and therefore, perturbation is likely to be especially important in species with stable social structures, like badgers, in which social bottlenecks are more likely to occur in the absence of perturbation.

From a management perspective, the considerable among‐individual variation in importance to transmission, together with the importance of indirect connections in the spatially structured networks, suggest that targeting individual badgers with high degree might not be the most effective strategy. It has been shown that when vaccination coverage is low, vaccination efforts that target betweenness centrality rather than degree, result in smaller epidemics (Rushmore et al., 2014; Salathé & Jones, 2010). However, our results suggest that in these networks (with intermediate levels of modularity), it is important to account for both degree and measures of indirect connections, such as betweenness and eigenvector centrality, when assessing the most important individuals for the spread of infection. A key challenge now is to identify those individual traits that relate to occupation of these network positions, in order to be able to target management interventions more efficiently (Delahay, Smith, & Hutchings, 2009; VanderWaal & Ezenwa, 2016). For example, in badgers, there is a tendency for individuals that use outlier setts (located away from the main setts), to occupy potentially important, bridging network positions with high numbers of direct and indirect connections (Weber, Carter, et al., 2013). Therefore, being able to target management interventions at outlier setts, or being able to better define seasonal variation in which individuals are likely to use these setts (Weber, Bearhop, et al., 2013) may contribute disproportionately to successful interventions. However, it has also been shown that when time is limited, it may be more effective to vaccinate many lower priority animals quickly, rather than waiting for opportunities to vaccinate more important individuals (Robinson et al., 2018).

In conclusion, using epidemiological simulations, we have shown that the stable social group structure of the European badger population manifests in modular contact networks that are likely to experience smaller epidemics than equivalent networks without this structure, especially for pathogens with intermediate transmission probabilities. The nature of these contact networks also means that it is important to take into account both direct and indirect connections of individuals in the network when determining their role in disease transmission, and that important individuals may differ for pathogens with different *R*_0_s. The design and implementation of effective disease management interventions should therefore acknowledge that individual variation in network positions, social groupings, and pathogen traits closely interact to influence transmission, and that the social systems of many wildlife populations might already be optimized for the containment or mitigation of the spread of disease.

## CONFLICT OF INTEREST

None declared.

## AUTHOR CONTRIBUTIONS

C.R., M.J.S., and M.B. conceived the idea for the manuscript. M.J.S. generated all of the networks used in the study as well as performed all statistical analysis. C.R. derived network measures and ran disease simulations. All authors contributed to the writing of successive drafts and all authors gave final approval for publication.

## DATA ACCESSIBILITY

The original weighted adjacency matrix for the high‐density population of European badgers, as well as code used for simulating networks and disease simulations can be found online https://doi.org/10.5061/dryad.49n3878.
