# Supplementary material for: Social structure contains epidemics and regulates individual roles in disease transmission in a group‐living mammal
Source: Ecol Evol. 2018 Nov 11;8(23):12044–55. doi: 10.1002/ece3.4664 (PMC6303749; doi:10.1002/ece3.4664)
Supplement: Supplementary file 1 [file ECE3-8-12044-s001.docx]

**Supplementary Material**

**Supplementary Tables**

**Table S1.** Information on the mean degree in unweighted versions of real and simulated networks at different levels of thresholding (filtering).

| Network | Mean degree | 2.5% quantile from simulations | Median from Simulations | 97.5% quantile from simulations |
| --- | --- | --- | --- | --- |
| No threshold (NF) | 9.69 | 8.86 | 9.65 | 10.43 |
| Threshold = 100s (F100) | 7.45 | 7.22 | 7.96 | 8.75 |
| Threshold =1000s (F1000) | 5.84 | 5.53 | 6.24 | 6.98 |

**Table S2.** R_0_ values along with interquartile ranges.

| **Transmission**  **Probability** | **SSN, SUN** | **RUN** |
| --- | --- | --- |
| 0.075 | 0.7342 (0.6777,0.7912) | 0.6847 (0.6665, 0.7030) |
| 0.1 | 0.9789 (0.9036,1.0549) | 0.9130 (0.8887, 0.9374) |
| 0.125 | 1.2236 (1.1295,1.3187) | 1.1412 (1.1108, 1.1717) |
| 0.15 | 1.4684 (1.3554,1.5824) | 1.3695 (1.3330, 1.4061) |
| 0.175 | 1.7131 (1.5812,1.8461) | 1.5977 (1.5551, 1.6404) |
| 0.2 | 1.9578 (1.8071,2.1099) | 1.8259 (1.7773, 1.8748) |
| 0.225 | 2.2026 (2.0330,2.3736) | 2.0542 (1.9995, 2.1091) |
| 0.25 | 2.4473 (2.2589,2.6374) | 2.2824 (2.2216, 2.3435) |
| 0.275 | 2.6920 (2.4848,2.9011) | 2.5107 (2.4438, 2.5778) |

Table S3. Epidemic threshold (T_C_) for the three graph types with different contact tolerance

| Network | No Filter (NF) | Filtered at 100s (F100) | Filtered at 1000s (F1000) |
| --- | --- | --- | --- |
| SSN/SUN | 0.10237 | 0.12412 | 0.15871 |
| RUN | 0.10957 | 0.13805 | 0.17532 |

**Table S4.** Spearman rank correlation measures (r_s_) for the relationship between network centrality measures and mean outbreak size in the three different network types used in the study (SSN = spatially structured network, SUN = spatially unstructured network and RUN = random network).

| Centrality measure | Transmission probability | SSN correlation | SUN correlation | RUN correlation |
| --- | --- | --- | --- | --- |
| Degree | 0.075 | 0.91 | 0.88 | 0.83 |
| Degree | 0.1 | 0.90 | 0.87 | 0.83 |
| Degree | 0.125 | 0.89 | 0.86 | 0.84 |
| Degree | 0.15 | 0.88 | 0.86 | 0.84 |
| Degree | 0.175 | 0.86 | 0.86 | 0.85 |
| Degree | 0.2 | 0.85 | 0.87 | 0.86 |
| Degree | 0.225 | 0.84 | 0.87 | 0.86 |
| Degree | 0.25 | 0.83 | 0.86 | 0.85 |
| Degree | 0.275 | 0.82 | 0.85 | 0.84 |
| Eigenvector centrality | 0.075 | 0.69 | 0.55 | -0.01 |
| Eigenvector centrality | 0.1 | 0.70 | 0.54 | -0.01 |
| Eigenvector centrality | 0.125 | 0.69 | 0.53 | 0.00 |
| Eigenvector centrality | 0.15 | 0.68 | 0.53 | 0.00 |
| Eigenvector centrality | 0.175 | 0.66 | 0.54 | -0.01 |
| Eigenvector centrality | 0.2 | 0.65 | 0.54 | -0.01 |
| Eigenvector centrality | 0.225 | 0.63 | 0.55 | 0.00 |
| Eigenvector centrality | 0.25 | 0.61 | 0.55 | -0.01 |
| Eigenvector centrality | 0.275 | 0.60 | 0.55 | 0.00 |
| Betweenness | 0.075 | 0.35 | 0.55 | 0.00 |
| Betweenness | 0.1 | 0.35 | 0.54 | 0.00 |
| Betweenness | 0.125 | 0.35 | 0.53 | 0.00 |
| Betweenness | 0.15 | 0.35 | 0.53 | 0.00 |
| Betweenness | 0.175 | 0.34 | 0.53 | -0.01 |
| Betweenness | 0.2 | 0.34 | 0.53 | -0.01 |
| Betweenness | 0.225 | 0.34 | 0.54 | 0.00 |
| Betweenness | 0.25 | 0.33 | 0.54 | -0.01 |
| Betweenness | 0.275 | 0.33 | 0.54 | 0.00 |

**Table S5.** Pseudo R-squared values for the linear and log-linear relationships between network centrality measures and mean outbreak size in the spatially structured network (SSN). Values are from linear mixed effects models where the response variable is the mean proportion of the population infected (logit transformed) and network identity has been fitted as a random effect.

| Centrality measure | Transmission probability | R squared  (linear fit) | R squared  (log-linear fit) |
| --- | --- | --- | --- |
| Degree | 0.075 | 0.83 | 0.85 |
| Degree | 0.1 | 0.81 | 0.85 |
| Degree | 0.125 | 0.79 | 0.84 |
| Degree | 0.15 | 0.77 | 0.84 |
| Degree | 0.175 | 0.76 | 0.83 |
| Degree | 0.2 | 0.75 | 0.83 |
| Degree | 0.225 | 0.74 | 0.83 |
| Degree | 0.25 | 0.73 | 0.82 |
| Degree | 0.275 | 0.71 | 0.81 |
| Eigenvector centrality | 0.075 | 0.51 | 0.54 |
| Eigenvector centrality | 0.1 | 0.53 | 0.56 |
| Eigenvector centrality | 0.125 | 0.54 | 0.57 |
| Eigenvector centrality | 0.15 | 0.53 | 0.57 |
| Eigenvector centrality | 0.175 | 0.53 | 0.56 |
| Eigenvector centrality | 0.2 | 0.52 | 0.55 |
| Eigenvector centrality | 0.225 | 0.51 | 0.54 |
| Eigenvector centrality | 0.25 | 0.49 | 0.53 |
| Eigenvector centrality | 0.275 | 0.47 | 0.51 |
| Betweenness | 0.075 | 0.11 | 0.11 |
| Betweenness | 0.1 | 0.12 | 0.13 |
| Betweenness | 0.125 | 0.14 | 0.14 |
| Betweenness | 0.15 | 0.16 | 0.16 |
| Betweenness | 0.175 | 0.18 | 0.18 |
| Betweenness | 0.2 | 0.19 | 0.19 |
| Betweenness | 0.225 | 0.19 | 0.20 |
| Betweenness | 0.25 | 0.19 | 0.20 |
| Betweenness | 0.275 | 0.19 | 0.20 |

**Table S6.** Measures of model fit using WAIC for seven potential models of the relationship between network type (SSN, SUN, RUN), transmission probability and repeatability in outbreak size. Lower WAIC indicates better model fit. All models used a log transformed response variable, assumed a Gaussian error distribution and employed weakly informative priors. The seven competing models were: 1: Log Repeatability ~ Network Type * Transmission Probability, 2: Log Repeatability ~ Network Type 2 * Transmission Probability, 3: Log Repeatability ~ Network Type 3 * Transmission Probability, 4: Log Repeatability ~ Transmission Probability, 5: Log Repeatability ~ Network Type, 6: Log Repeatability ~ Network Type 2, 7: Log Repeatability ~ Network Type 3. Transmission probability was fitted as a 9-level factor. Network Type was a 3-level factor that distinguished between SSN, SUN and RUN networks. Network type 2 was a 2-level factor that distinguished between non-random (SSN and SUN) and random networks. Network type 3 was a 2-level factor that distinguished between spatially structured (SSN) and spatially unstructured (SUN and RUN) networks.

| Model Comparison | No threshold | Threshold = 100 | Threshold = 1000 |
| --- | --- | --- | --- |
| Model 1 (full) | **15410±307** | 85792±201 | 91812±161 |
| Model 2 (non-random vs random) | 21770±274 | 86100±202 | 91940±162 |
| Model 3 (spatial vs non-spatial) | 26542±288 | 86480±204 | 92248±162 |
| Model 4 (no effect of network type) | 39974±246 | 87718±210 | 92902±166 |
| Model 5 (no effect of transmission probability – SSN vs. SUN vs. RUN) | 74846±262 | **28866±217** | **91765±160** |
| Model 6 (no effect of transmission probability – random vs. non-random) | 75572±259 | 86068±202 | 91908±162 |
| Model 7 (no effect of transmission probability – spatial vs. non-spatial) | 76275±267 | 86449±204 | 92216±162 |

**Table S7.** Measures of model fit (see Table S6) using WAIC for seven potential models of the relationship between network type, transmission probability and repeatability in the number of secondary infections (as per above we only conducted this analysis for networks with no threshold). Lower WAIC indicates better model fit.

| **Model Comparison** | **No threshold** |
| --- | --- |
| Model 1 (full) | 28913±217 |
| Model 2 (non-random vs random) | 28896±217 |
| Model 3 (spatial vs non-spatial) | 33744±225 |
| Model 4 (no effect of network type) | 35145±228 |
| Model 5 (no effect of transmission probability – SSN vs. SUN vs. RUN) | 28865±217 |
| Model 6 (no effect of transmission probability – random vs. non-random) | **28864±217** |
| Model 7 (no effect of transmission probability – spatial vs. non-spatial) | 33713±225 |

**Table S8**. Parameter estimates from model 1 (the full model) for the relationship between network type, transmission probability and repeatability in outbreak size for networks at three different levels of thresholding used in the study.

| **Parameter** | **No threshold** | **Threshold = 100** | **Threshold = 1000** |
| --- | --- | --- | --- |
| Intercept (Random/0.075) | -6.93±0.01 | -4.66±0.04 | -4.87±0.04 |
| Type - Structured | 1.13±0.01 | 0.79±0.05 | 0.67±0.06 |
| Type - Unstructured | 0.78±0.01 | 0.46±0.05 | 0.43±0.06 |
| T - 0.1 | 1.14±0.01 | -0.01±0.05 | 0.01±0.06 |
| T - 0.125 | 2.03±0.01 | -0.01±0.05 | 0.01±0.06 |
| T - 0.15 | 2.60±0.01 | 0.00±0.05 | 0.02±0.06 |
| T - 0.175 | 2.86±0.01 | -0.01±0.05 | 0.01±0.06 |
| T - 0.2 | 2.97±0.01 | -0.01±0.05 | 0.01±0.06 |
| T - 0.225 | 2.96±0.01 | -0.01±0.05 | 0.00±0.06 |
| T - 0.25 | 2.85±0.01 | 0.00±0.05 | 0.01±0.06 |
| T - 0.275 | 2.64±0.01 | -0.00±0.05 | 0.01±0.06 |
| Structured : 0.1 | -0.17±0.02 | 0.01±0.07 | -0.01±0.08 |
| Unstructured : 0.1 | -0.08±0.02 | 0.02±0.08 | -0.01±0.08 |
| Structured : 0.125 | -0.28±0.02 | -0.00±0.08 | -0.01±0.08 |
| Unstructured : 0.125 | -0.17±0.02 | 0.01±0.08 | -0.01±0.08 |
| Structured : 0.15 | -0.31±0.02 | 0.00±0.07 | -0.01±0.08 |
| Unstructured : 0.15 | -0.25±0.02 | 0.01±0.08 | -0.02±0.08 |
| Structured : 0.175 | -0.26±0.02 | 0.01±0.08 | -0.01±0.08 |
| Unstructured : 0.175 | -0.28±0.02 | 0.02±0.07 | -0.01±0.08 |
| Structured : 0.2 | -0.24±0.02 | -0.00±0.08 | -0.01±0.08 |
| Unstructured : 0.2 | -0.31±0.02 | 0.01±0.08 | -0.01±0.08 |
| Structured : 0.225 | -0.20±0.02 | 0.01±0.08 | 0.00±0.08 |
| Unstructured : 0.225 | -0.32±0.02 | 0.02±0.08 | -0.01±0.08 |
| Structured : 0.25 | -0.14±0.02 | -0.00±0.07 | -0.01±0.08 |
| Unstructured : 0.25 | -0.31±0.02 | 0.00±0.08 | -0.02±0.08 |
| Structured : 0.275 | -0.07±0.02 | -0.01±0.08 | -0.01±0.08 |
| Unstructured : 0.275 | -0.28±0.02 | 0.01±0.08 | -0.02±0.08 |

**Supplementary Figures**


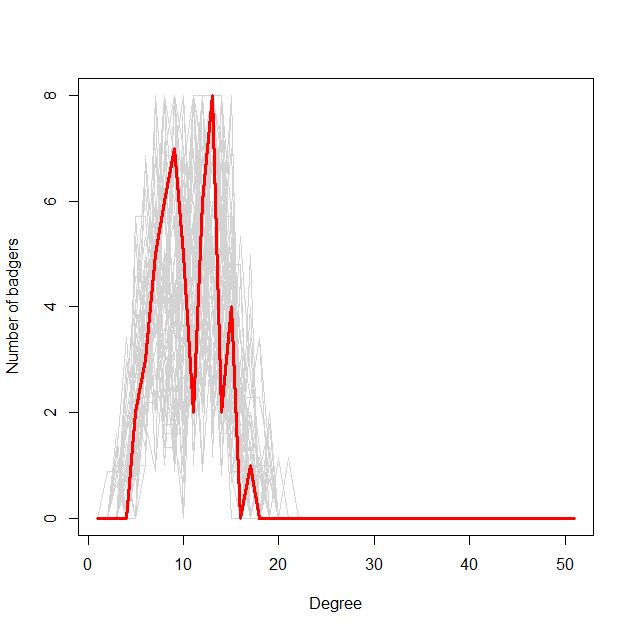


**Figure S1.** Degree distribution in the observed network in red, and in 50 example simulated networks (in light grey) for the unweighted full network (no threshold).

**
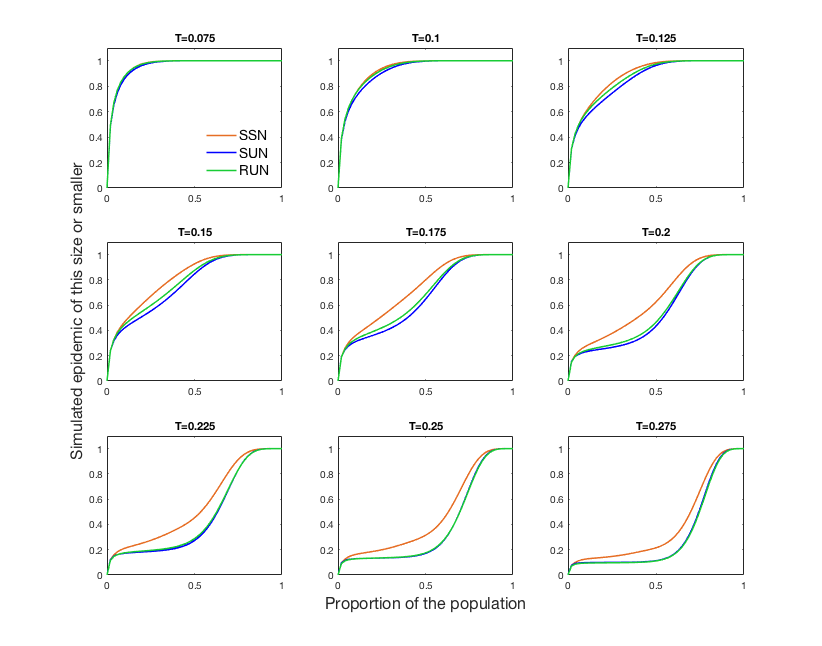
**

**Figure S2**. Cumulative simulated epidemics size as a proportion of the population for spatially structured networks (orange), spatially unstructured networks (blue) and random network (green) for 9 different transmission probabilities (T=0.075-0.275). Smaller outbreaks are more frequent if lines are higher for smaller proportions of the population. The figure represents combined data from all 5,100,000 simulated epidemics for each transmission probability.


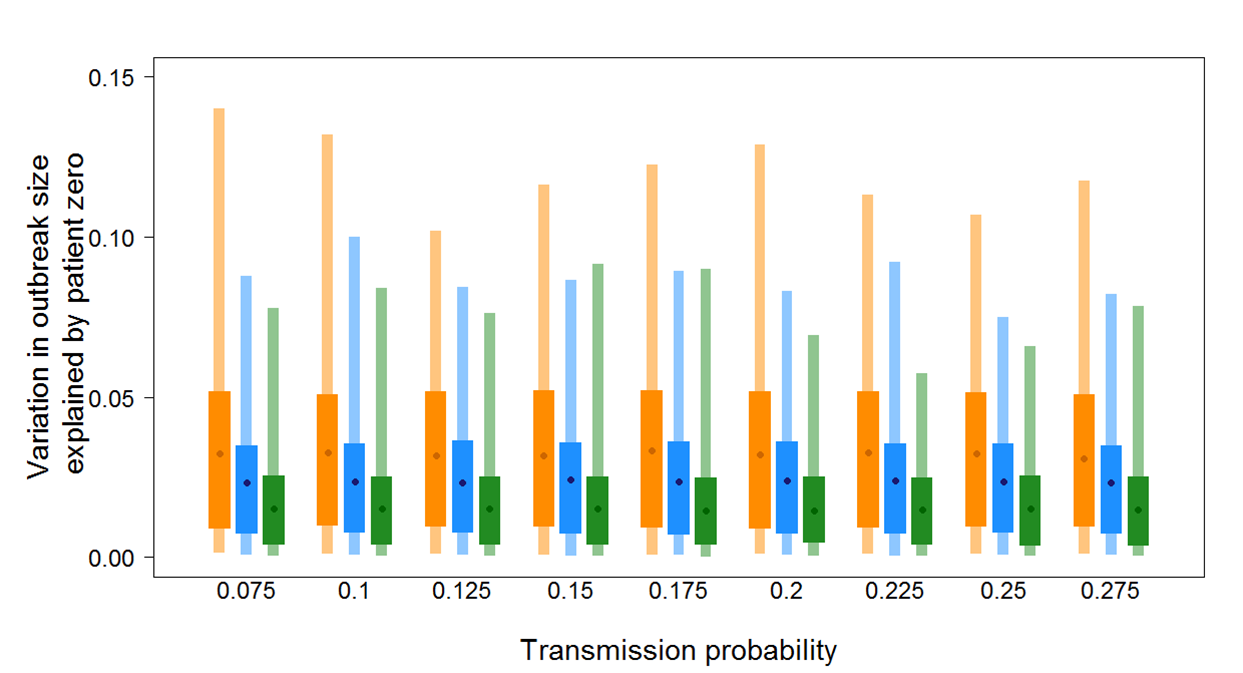


**Figure S3.** The proportion of variation in the outbreak size explained by the choice of the initially infected individual (patient zero) in networks filtered to retain only contacts with a strength greater than 100 seconds (F100). Orange is the structured network (SSN), blue is the unstructured network (SUN) and green is the random network (RUN). Points represent the median, wide boxes the interquartile range and narrow boxes the range of values calculated from each of the 1000 simulated networks of each type.


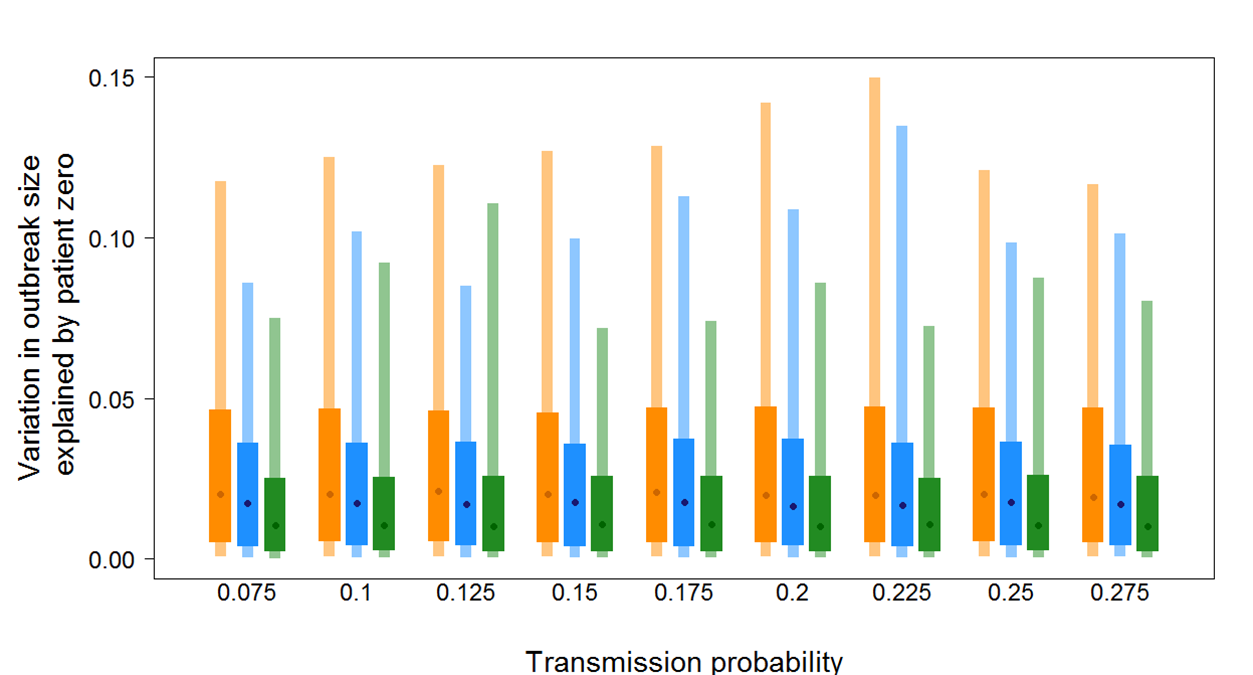


**Figure S4.** The proportion of variation in the outbreak size explained by the choice of the initially infected individual (patient zero) in networks filtered to retain only contacts with a strength greater than 1000 seconds (F1000). Orange is the structured network (SSN), blue is the unstructured network (SUN) and green is the random network (RUN). Points represent the median, wide boxes the interquartile range and narrow boxes the range of values calculated from each of the 1000 simulated networks of each type.
