# Supplementary material for: Social structure contains epidemics and regulates individual roles in disease transmission in a group‐living mammal
Source: Ecol Evol. 2018 Nov 11;8(23):12044–55. doi: 10.1002/ece3.4664 (PMC6303749; doi:10.1002/ece3.4664)
Supplement: Supplementary file 2 [file ECE3-8-12044-s002.docx]

**R Code for network generation simulations**

###-----------------------------

##1. set directory

###-------------------------------

##2. Load required R packages

require(MASS)

require(squash)

require(LaplacesDemon)

require(pscl)

require(emdbook)

require(igraph)

###-------------------------------

###-------------------------------

#3. Fit models to observed network

#read in and set up matrix

mat<-read.csv("Network.csv")

names<-mat[,1]

mat<-as.matrix(mat[,2:ncol(mat)])

rownames(mat)<-colnames(mat)<-names

#read in badger locations

locs<-read.csv("Locations.csv")

#read in group information

sogrs<-read.csv("Groups.csv")

spgrs<-read.csv("SpatGroups.csv")

#sett up a distance between territories matrix

#rows/colums correspond to spatial group numbers

terr.mat<-rbind(c(0,1,3,1,1,1,2,2),c(1,0,4,2,1,2,3,3),c(3,4,0,4,3,2,5,1),c(1,2,4,0,2,2,1,2),c(1,1,3,2,0,1,3,2),c(1,2,2,2,1,0,3,1),c(2,3,5,1,3,3,0,4),c(2,3,1,2,2,1,4,0))

#-------------------------------------------------

#just upper triangle of matrix

mat.2<-mat[upper.tri(mat)]

#create distance matrix from sett location information

distmat<-as.matrix(dist(locs[,3:4]))

rownames(distmat)<-colnames(distmat)<-names

distmat2<-distmat[upper.tri(distmat)]

groupmat<-array(NA,dim=rep(nrow(mat),2))

for(i in 1:nrow(groupmat)){

for(j in 1:nrow(groupmat)){

ifelse(spgrs[i,2]==spgrs[j,2],groupmat[i,j]<-1,groupmat[i,j]<-0)

}

}

groupmat2<-groupmat[upper.tri(groupmat)]

terrdist<-array(NA,dim=rep(nrow(mat),2))

for(i in 1:nrow(groupmat)){

for(j in 1:nrow(groupmat)){

terrdist[i,j]<-terr.mat[spgrs[i,2],spgrs[j,2]]

}

}

terrdist2<-terrdist[upper.tri(terrdist)]

#fit zero-inflated negative binomial model to data

modtot<-zeroinfl(mat.2~terrdist2+distmat2+groupmat2,dist="negbin")

#extract model coefficients

bin.coefs<--summary(modtot)$coefficients$zero[,1]

count.coefs<-summary(modtot)$coefficients$count[,1]

###-------------------------------

###-------------------------------

#3. Simulate raw network data

#setup necessary parameters

#re-generate dataframe equivalent to real data

locs2<-data.frame(locs,spgrs[,2])

terr.mat<-terr.mat

indiv.info<-data.frame(locs2[,c(1,3,4,5)])

names(indiv.info)<-c("ID","X","Y","Gr")

#--------------------------------

#4. generate 10000 networks

#array to store results

nets.100<-array(NA,dim=c(rep(nrow(indiv.info),2),10000))

for (n in 1:10000){

matrix<-array(NA,dim=rep(nrow(indiv.info),2))

colnames(matrix)<-rownames(matrix)<-indiv.info[,1]

for(i in 1:(nrow(matrix)-1)){

for(j in (i+1):nrow(matrix)){

dista<-as.vector(dist(indiv.info[c(i,j),2:3]))

Ga<-NA

ifelse(indiv.info[i,4]==indiv.info[j,4],Ga<-1,Ga<-0)

Ta<-terr.mat[indiv.info[i,4],indiv.info[j,4]]

pred<-exp(bin.coefs[1]+Ta*bin.coefs[2]+dista*bin.coefs[3]+Ga*bin.coefs[4])/(1+exp(bin.coefs[1]+Ta*bin.coefs[2]+dista*bin.coefs[3]+Ga*bin.coefs[4]))

tmp<-rbern(1,pred)

if(tmp==1){

res<-c(exp(count.coefs[1]+Ta*count.coefs[2]+dista*count.coefs[3]+Ga*count.coefs[4]),exp(count.coefs[5]))

matrix[i,j]<-rnbinom(1,mu=res[1],size=res[2])

}

else{

matrix[i,j]<-0

}

}

}

for(i in 1:(nrow(matrix)-1)){

for(j in (i+1):nrow(matrix)){

matrix[j,i]<-matrix[i,j]

}

}

diag(matrix)<-0

nets.100[,,n]<-matrix

print(paste(n))

}

###-------------------------------

###-------------------------------

#5. generate binary (non-filtered) networks

nets.100.unw<-sign(nets.100)

#calculate degrees

deg.nets<-apply(nets.100.unw,3,colSums)

deg.dists<-matrix(NA,nr=nrow(indiv.info),nc=10000)

rownames(deg.dists)<-seq(0,50,1)

colnames(deg.dists)<-seq(1,10000,1)

for(i in 1:10000){

for(j in 1:nrow(deg.dists)){

deg.dists[j,i]<-sum(deg.nets[,i]==(j-1))/nrow(indiv.info)

}

}

deg.means<-apply(deg.nets,2,mean)

ex.deg.dists<-matrix(NA,nr=nrow(indiv.info)-1,nc=10000)

for(i in 1:10000){

for(j in 1:(nrow(deg.dists)-1)){

ex.deg.dists[j,i]<-(j*deg.dists[(j+1),i])/deg.means[i]

}

}

rownames(ex.deg.dists)<-seq(0,49,1)

colnames(ex.deg.dists)<-seq(1,10000,1)

###-------------------------------

###-------------------------------

#6. create F100 filtered networks

nets.100.T1<-nets.100-100

re.zero<-function(a){

if(a<1){a<-0}else{a<-1}

}

nets.100.T1.unw<-apply(nets.100.T1,1:3,re.zero)

#calculate degrees

deg.nets.T1<-apply(nets.100.T1.unw,3,colSums)

deg.dists.T1<-matrix(NA,nr=nrow(indiv.info),nc=10000)

rownames(deg.dists.T1)<-seq(0,50,1)

colnames(deg.dists.T1)<-seq(1,10000,1)

for(i in 1:10000){

for(j in 1:nrow(deg.dists.T1)){

deg.dists.T1[j,i]<-sum(deg.nets.T1[,i]==(j-1))/nrow(indiv.info)

}

}

deg.means.T1<-apply(deg.nets.T1,2,mean)

ex.deg.dists.T1<-matrix(NA,nr=nrow(indiv.info)-1,nc=10000)

for(i in 1:10000){

for(j in 1:(nrow(deg.dists.T1)-1)){

ex.deg.dists.T1[j,i]<-(j*deg.dists.T1[(j+1),i])/deg.means.T1[i]

}

}

rownames(ex.deg.dists.T1)<-seq(0,49,1)

colnames(ex.deg.dists.T1)<-seq(1,10000,1)

###-------------------------------

###-------------------------------

#7. generate F1000 networks

nets.100.T2<-nets.100-1000

re.zero<-function(a){

if(a<1){a<-0}else{a<-1}

}

nets.100.T2.unw<-apply(nets.100.T2,1:3,re.zero)

#calculate degrees

deg.nets.T2<-apply(nets.100.T2.unw,3,colSums)

deg.dists.T2<-matrix(NA,nr=nrow(indiv.info),nc=10000)

rownames(deg.dists.T2)<-seq(0,50,1)

colnames(deg.dists.T2)<-seq(1,10000,1)

for(i in 1:10000){

for(j in 1:nrow(deg.dists.T2)){

deg.dists.T2[j,i]<-sum(deg.nets.T2[,i]==(j-1))/nrow(indiv.info)

}

}

deg.means.T2<-apply(deg.nets.T2,2,mean)

ex.deg.dists.T2<-matrix(NA,nr=nrow(indiv.info)-1,nc=10000)

for(i in 1:10000){

for(j in 1:(nrow(deg.dists.T2)-1)){

ex.deg.dists.T2[j,i]<-(j*deg.dists.T2[(j+1),i])/deg.means.T2[i]

}

}

rownames(ex.deg.dists.T2)<-seq(0,49,1)

colnames(ex.deg.dists.T2)<-seq(1,10000,1)

###-------------------------------

###-------------------------------

#8. calculate same data from observed matrix for better checking

mat.B<-sign(mat)

ds.o<-colSums(mat.B)

d.m.o<-mean(ds.o)

d.d.o<-rep(NA,nrow(indiv.info))

for(i in 1:length(d.d.o)){

d.d.o[i]<-sum(ds.o==(i-1))/51

}

names(d.d.o)<-seq(0,50,1)

ex.d.o<-rep(NA,(nrow(indiv.info)-1))

for(i in 1:length(ex.d.o)){

ex.d.o[i]<-(i*d.d.o[(i+1)])/d.m.o

}

#---------------------

matB2<-sign(mat-100)

for(i in 1:length(matB2)){

if(matB2[i]<0){matB2[i]<-0}

}

ds.o.T1<-colSums(matB2)

d.m.o.T1<-mean(ds.o.T1)

#----------------------

matB3<-sign(mat-1000)

for(i in 1:length(matB3)){

if(matB3[i]<0){matB3[i]<-0}

}

ds.o.T2<-colSums(matB3)

d.m.o.T2<-mean(ds.o.T2)

#----------------------

dev.new()

par(mfrow=c(3,3))

plot(d.d.o,type="l")

plot(deg.dists[,1],type="l")

plot(deg.dists[,2],type="l")

plot(deg.dists[,3],type="l")

plot(deg.dists[,4],type="l")

plot(deg.dists[,5],type="l")

plot(deg.dists[,6],type="l")

plot(deg.dists[,7],type="l")

plot(deg.dists[,8],type="l")

dev.new()

par(mfrow=c(3,3))

plot(ex.d.o,type="l")

plot(ex.deg.dists[,1],type="l")

plot(ex.deg.dists[,2],type="l")

plot(ex.deg.dists[,3],type="l")

plot(ex.deg.dists[,4],type="l")

plot(ex.deg.dists[,5],type="l")

plot(ex.deg.dists[,6],type="l")

plot(ex.deg.dists[,7],type="l")

plot(ex.deg.dists[,8],type="l")

hist(deg.means)

hist(deg.means.T1)

hist(deg.means.T2)

d.m.o.T2

###-------------------------------

###-------------------------------

#9. produce spatially unstructured non-filtered network

nets.100.n<-array(NA,dim=dim(nets.100))

dev.new()

par(mfrow=c(5,2),mar=rep(0,4))

for(i in 1:10000){

g<-graph.adjacency(sign(nets.100[,,i]),mode="undirected")

g2<-rewire(g,keeping_degseq(loops=F,1000))

nets.100.n[,,i]<-as.matrix(as_adj(g2))

}

###-------------------------------

###-------------------------------

#10. produce spatially unstructured F100 network

nets.100.T1.n<-array(NA,dim=dim(nets.100))

dev.new()

par(mfrow=c(5,2),mar=rep(0,4))

for(i in 1:10000){

g<-graph.adjacency(sign(nets.100.T1.unw[,,i]),mode="undirected")

g2<-rewire(g,keeping_degseq(loops=F,1000))

nets.100.T1.n[,,i]<-as.matrix(as_adj(g2))

}

###-------------------------------

###-------------------------------

#11. produce spatially unstructured F1000 network

nets.100.T2.n<-array(NA,dim=dim(nets.100))

dev.new()

par(mfrow=c(5,2),mar=rep(0,4))

for(i in 1:10000){

g<-graph.adjacency(sign(nets.100.T2.unw[,,i]),mode="undirected")

g2<-rewire(g,keeping_degseq(loops=F,1000))

nets.100.T2.n[,,i]<-as.matrix(as_adj(g2))

}

###-------------------------------

###-------------------------------

#12. Simulate random networks

###set directory

#------------------------

###set seed

set.seed(3)

#load required R package

require(igraph)

Rn<-Rn.100<-Rn.1000<-array(NA,dim=c(51,51,10000))

for(i in 1:10000){

g<-erdos.renyi.game(51,p.or.m=238,type="gnm",mode="undirected",loops=FALSE)

g.100<-erdos.renyi.game(51,p.or.m=189,type="gnm",mode="undirected",loops=FALSE)

g.1000<-erdos.renyi.game(51,p.or.m=150,type="gnm",mode="undirected",loops=FALSE)

Rn[,,i]<-as.matrix(as_adj(g))

Rn.100[,,i]<-as.matrix(as_adj(g.100))

Rn.1000[,,i]<-as.matrix(as_adj(g.1000))

}

for(i in 1:10000){

write.csv(Rn[,,i],paste0("rNT_",i,".csv"))

write.csv(Rn.100[,,i],paste0("rT100_",i,".csv"))

write.csv(Rn.1000[,,i],paste0("rT1000_",i,".csv"))

}

**R Code for calculating repeatability in outbreak size**

###set directory

#-----------------

###load R packages

library(rptR)

library(lme4)

#-----------------

###The data required for this analysis are big dataframes of outbreak size for each run of the simulation.

###The data generated from our simulated networks are also provided

str<-as.matrix(read.csv("SN.csv",header=FALSE))

struc<-c(str)

ids<-rep(rep(1:51,each=100),length(struc)/5100)

net<-rep(1:1000,each=nrow(str))

fail<-51-struc

t<-rep(rep(seq(0.075,0.275,by=0.025),each=5100),1000)

str.fr<-data.frame(ids,net,t,struc,fail)

t2<-seq(0.075,0.275,by=0.025)

rpts<-matrix(NA,nr=1000,nc=9)

for(i in 1:1000){

for(j in 1:9){

model<-model<-rptProportion(cbind(struc, fail) ~ (1|ids),grname=c("ids"), data=str.fr[str.fr$net==i&str.fr$t==t2[j],],npermut=0,nboot=0)

rpt<-as.vector(model$R[1,1])

rpts[i,j]<-rpt

print(paste(i,"-",j))

}

}

#--------------------------------------

un<-as.matrix(read.csv("UN.csv",header=FALSE))

unstruc<-c(un)

ids<-rep(rep(1:51,each=100),length(unstruc)/5100)

net<-rep(1:1000,each=nrow(un))

fail<-51-unstruc

t<-rep(rep(seq(0.075,0.275,by=0.025),each=5100),1000)

unstr.fr<-data.frame(ids,net,t,unstruc,fail)

rpts.u<-matrix(NA,nr=1000,nc=9)

for(i in 1:1000){

for(j in 1:9){

model<-model<-rptProportion(cbind(unstruc, fail) ~ (1|ids),grname=c("ids"), data=unstr.fr[unstr.fr$net==i&unstr.fr$t==t2[j],],npermut=0,nboot=0)

rpt<-as.vector(model$R[1,1])

rpts.u[i,j]<-rpt

print(paste(i,"-",j))

}

}

#----------------------------------------

rn<-as.matrix(read.csv("RN.csv",header=FALSE))

random<-c(rn)

ids<-rep(rep(1:51,each=100),length(random)/5100)

net<-rep(1:1000,each=nrow(rn))

fail<-51-random

t<-rep(rep(seq(0.075,0.275,by=0.025),each=5100),1000)

rn.fr<-data.frame(ids,net,t,random,fail)

library(lme4)

rpts.r<-matrix(NA,nr=1000,nc=9)

for(i in 1:1000){

for(j in 1:9){

model<-model<-rptProportion(cbind(random, fail) ~ (1|ids),grname=c("ids"), data=rn.fr[rn.fr$net==i&rn.fr$t==t2[j],],npermut=0,nboot=0)

rpt<-as.vector(model$R[1,1])

rpts.r[i,j]<-rpt

print(paste(i,"-",j))

}

}

write.csv(rpts,"struc_rpts.csv")

write.csv(rpts.u,"unstruc_rpts.csv")

write.csv(rpts.r,"random_rpts.csv")

**R Code for calculating repeatability in the number of secondary infections**

###set directory

#-----------------

###load R packages

library(rptR)

library(lme4)

#-----------------

###The data required for this analysis are big dataframes of the number of secondary infections for each run of the simulation.

###The data generated from our simulated networks are also provided

str<-as.matrix(read.csv("Ro_SN.csv",header=FALSE))

struc<-c(str)

ids<-rep(rep(1:51,each=100),length(struc)/5100)

net<-rep(1:1000,each=nrow(str))

fail<-51-struc

t<-rep(rep(seq(0.075,0.275,by=0.025),each=5100),1000)

str.fr<-data.frame(ids,net,t,struc,fail)

library(lme4)

t2<-seq(0.075,0.275,by=0.025)

rpts<-matrix(NA,nr=1000,nc=9)

for(i in 1:1000){

for(j in 1:9){

model<-model<-rptPoisson(struc ~ (1|ids),grname=c("ids"), data=str.fr[str.fr$net==i&str.fr$t==t2[j],],npermut=0,nboot=0)

rpt<-as.vector(model$R[1,1])

rpts[i,j]<-rpt

print(paste(i,"-",j))

}

}

#-----------------

un<-as.matrix(read.csv("Ro_UN.csv",header=FALSE))

unstruc<-c(un)

ids<-rep(rep(1:51,each=100),length(unstruc)/5100)

net<-rep(1:1000,each=nrow(un))

fail<-51-unstruc

t<-rep(rep(seq(0.075,0.275,by=0.025),each=5100),1000)

unstr.fr<-data.frame(ids,net,t,unstruc,fail)

library(lme4)

rpts.u<-matrix(NA,nr=1000,nc=9)

for(i in 1:1000){

for(j in 1:9){

model<-model<-rptPoisson(unstruc ~ (1|ids),grname=c("ids"), data=unstr.fr[unstr.fr$net==i&unstr.fr$t==t2[j],],npermut=0,nboot=0)

rpt<-as.vector(model$R[1,1])

rpts.u[i,j]<-rpt

print(paste(i,"-",j))

}

}

#-----------------

rn<-as.matrix(read.csv("Ro_RN.csv",header=FALSE))

random<-c(rn)

ids<-rep(rep(1:51,each=100),length(random)/5100)

net<-rep(1:1000,each=nrow(rn))

fail<-51-random

t<-rep(rep(seq(0.075,0.275,by=0.025),each=5100),1000)

rn.fr<-data.frame(ids,net,t,random,fail)

rpts.r<-matrix(NA,nr=1000,nc=9)

for(i in 1:1000){

for(j in 1:9){

model<-model<-rptPoisson(random ~ (1|ids),grname=c("ids"), data=rn.fr[rn.fr$net==i&rn.fr$t==t2[j],],npermut=0,nboot=0)

rpt<-as.vector(model$R[1,1])

rpts.r[i,j]<-rpt

print(paste(i,"-",j))

}

}

write.csv(rpts,"struc_R0_rpts.csv")

write.csv(rpts.u,"unstruc_R0_rpts.csv")

write.csv(rpts.r,"random_R0_rpts.csv")

**MATLAB code for running disease outbreak**

% This scrip simulated disease spread on network A, the binary adjacency

% matrix and phi is the transmission prob, In is initially infected.

function Cluster=Simulation(A,phi,In)

% A is the adjacency matrix.

n=length(A); %number of badgers in the network

Outbreak=zeros(1,n); %this keeps track of all individulas that have been invected.

Outbreak(In)=1; % account for the initial infected

%B gives the total epidemic size

B=zeros(n,n);%contains all iterations

B(1,In)=1;

for i=1:n-1%n-1

Bn=B(i,:)*A;

Bn=Bn>0; % turns the double links into 1s

Bn=Bn-Outbreak;% deletes nodes that have already been ifected or makes it -1

Nn=Bn<0;

Bn=Bn+Nn;

for j=1:length(Bn)

Bn(j)=Bn(j)*(sum(rand >= cumsum([0, 1-phi, phi]))-1); % applies prob of transmission

end

B(i+1,:)=Bn;

Outbreak=Outbreak+Bn;

end

%Cluster=Outbreak

Cluster=sum(Outbreak);
